# Programmable Base Editing of the Sheep Genome Revealed No Genome-Wide Off-Target Mutations

**DOI:** 10.3389/fgene.2019.00215

**Published:** 2019-03-15

**Authors:** Shiwei Zhou, Bei Cai, Chong He, Ying Wang, Qiang Ding, Jiao Liu, Yao Liu, Yige Ding, Xiaoe Zhao, Guanwei Li, Chao Li, Honghao Yu, Qifang Kou, Wenzhi Niu, Bjoern Petersen, Tad Sonstegard, Baohua Ma, Yulin Chen, Xiaolong Wang

**Affiliations:** ^1^College of Animal Science and Technology, Northwest A&F University, Yangling, China; ^2^College of Information Engineering, Northwest A&F University, Yangling, China; ^3^College of Veterinary Medicine, Northwest A&F University, Yangling, China; ^4^Guilin Medical University, Guilin, China; ^5^Ningxia Tianyuan Tan Sheep Farm, Hongsibu, China; ^6^Institute of Farm Animal Genetics, Friedrich-Loeffler-Institut, Neustadt, Germany; ^7^Recombinetics, Saint Paul, MN, United States

**Keywords:** base editing, genome editing, point mutation, whole genome sequencing, off-target mutation

## Abstract

Since its emergence, CRISPR/Cas9-mediated base editors (BEs) with cytosine deaminase activity have been used to precisely and efficiently introduce single-base mutations in genomes, including those of human cells, mice, and crop species. Most production traits in livestock are induced by point mutations, and genome editing using BEs without homology-directed repair of double-strand breaks can directly alter single nucleotides. The p.96R > C variant of Suppressor cytokine signaling 2 (SOCS2) has profound effects on body weight, body size, and milk production in sheep. In the present study, we successfully obtained lambs with defined point mutations resulting in a p.96R > C substitution in *SOCS2* by the co-injection of BE3 mRNA and a single guide RNA (sgRNA) into sheep zygotes. The observed efficiency of the single nucleotide exchange in newborn animals was as high as 25%. Observations of body size and body weight in the edited group showed that gene modification contributes to enhanced growth traits in sheep. Moreover, targeted deep sequencing and unbiased family trio-based whole genome sequencing revealed undetectable off-target mutations in the edited animals. This study demonstrates the potential for the application of BE-mediated point mutations in large animals for the improvement of production traits in livestock species.

## Introduction

Clustered regularly interspaced short palindromic repeat (CRISPR)/CRISPR-associated (Cas) 9 is widely used to establish site-specific genome-edited cell lines and animal models (Sander and Joung, 2014). The Cas9 protein, under the guidance of single guide (sg) RNA, cleaves DNA at sequence-specific sites in the genome and produces a double-strand break (DSB). To response DSB, cellular DNA repair pathways generated more abundant insertion and deletions (indels) by non-homologous end-joining (NHEJ) than that of homology-directed repair (HDR)-mediated gene correction (Chu et al., 2015; Paquet et al., 2016; Sakuma et al., 2016). Therefore, developing alternative approaches to correct point mutations that do not need DSBs is highly expected. Rat cytidine deaminase (rAPOBEC1) linked to nCas9 (Cas9 nickase) was reported to efficiently convert C→T at target sites without introducing DSBs (Komor et al., 2016). After several generations of modification, base editor 3 (BE3) including rAPOBEC1, nCas9 (A840H), and uracil DNA glycosylase inhibitor (UGI) was developed; the mutation efficiency was up to 74.9% in mammalian cells (Komor et al., 2016). To further optimize BE3, previous studies have been conducted to improve target specificity (Kim D. et al., 2017), editing efficiency and product purity (Komor et al., 2017), expand the genome-targeting scope (Kim K. et al., 2017), and reduce off-target effects (Kim D. et al., 2017). To prove that BE3 has a high efficiency for converting C:G to T:A base pairs, several groups have used BE3 to silence genes by introducing nonsense mutations (Billon et al., 2017; Kuscu et al., 2017).

Sheep are a phenotypically diverse livestock species that are raised globally for meat, milk, and fiber production. Suppressor of cytokine signaling 2 (SOCS2), a member of the SOCS protein family, is a negative regulator of biological processes mediated by various cytokines, such as metabolism, skeletal muscle development, and the response to infection (Inagaki-Ohara et al., 2014; Letellier and Haan, 2016). The most important of these processes is the regulation of GH signaling during growth and development (Yang et al., 2012; Dobie et al., 2018). *SOCS2* is the major gene involved in the promotion of bone development in mice, and it plays a vital role in the control of bone mass and body weight (Metcalf et al., 2000; Dobie et al., 2018). A point mutation g.C1901T (p.R96C) in *SOCS2* that completely abrogates *SOCS2* binding affinity for the phosphopeptide of growth hormone receptor (GHR) is highly associated with an increased body weight and size in sheep (Rupp et al., 2015). We recently reported the usage of the BE3 system to induce nonsense mutations in the goat *FGF5* gene, to generate animals with longer hair fibers (Li G. et al., 2018). It was the first base editing study in large animals and further inspired us to examine the feasibility of induce amino acid exchanges in sheep. In the present study, we obtained BE3-mediated lambs by co-injection of a BE3 mRNA and guide RNA target the p.R96C variant in *SOCS2*. In addition, we used a parent-progeny whole genome sequencing (WGS) approach to show that no off-target mutations were detected and the mutation frequency in edited animals is equivalent to that in control groups.

## Materials and Methods

### Animals

Tan sheep were maintained at the Ningxia Tianyuan Sheep Farm, Hongsibu, Ningxia Autonomous Region, China. All experimental animals were provided water and standard feed *ad libitum*, consistent with normal sheep, and were treated according to Guidelines for the Care and Use of Laboratory Animals formulated by the College of Animal Science and Technology, Northwest A&F University. The experimental study was approved by the Northwest A&F University Animal Care and Use Committee (Approval ID: 2016NXTS001).

### Design of sgRNA

The sequences target the g.C1901T (p.R96C) variant in the ovine *SOCS2* gene is listed in Supplementary Table S1. Two oligonucleotides (Supplementary Table S2) used for the transcription of sgRNA *in vitro* were precisely synthesized and annealed to form double-stranded oligos. These double-stranded oligos were subcloned into the pUC57-T7-gRNA vector as described previously (Shen et al., 2013). The clones containing the desired sequence were selected, expanded by cultivation, and the plasmid was extracted using a plasmid extraction kit (AP-MN-P-250G; Axygen, Union City, CA, United States), sgRNA was transcribed *in vitro* using the MEGAshortscript Kit (AM1354; Ambion, Foster City, CA, United States) and purified using the MEGAclear Kit (AM1908; Ambion). Subsequently, the BE3 mRNA *in vitro* transcription vector (No. 44758; Addgene, Cambridge, MA, United States) was used as a template to produce BE3 mRNAs following a previously published protocol (Shen et al., 2013).

### Production of Single-Nucleotide Mutation Sheep

Healthy ewes (3–5 years old) with regular estrous cycles were selected as donors for zygote collection. The superovulation treatment of donors and the procedures for zygote collection were as described previously (Wang et al., 2015). Briefly, an EAZI-BREED controlled internal drug release (CIDR) Sheep and Goat Device (containing 300 mg of progesterone) was inserted into the vagina of the donor sheep for 12 days and superovulation was performed 60 h before CIDR Device removal. Zygotes at the 1-cell stage were surgically collected and immediately transferred to TCM-199 medium (Gibco, Gaithersburg, MD, United States). A mixture of BE3 mRNA (25 ng μL^-1^) and sgRNA (10 ng μL^-1^) was co-injected into the cytoplasm of 1-cell stage zygotes using an Eppendorf FemtoJet system. The injection pressure, injection time, and compensatory pressure were 45 kPa, 0.1 s, and 7 kPa, respectively. Microinjections were performed on the heated stage of the Olympus ON3 micromanipulation system. Injected embryos were cultured in Quinn’s Advantage Cleavage Medium and Blastocyst Medium (Sage BioPharma, Toronto, ON, Canada) for ∼24 h and were then transferred into surrogates, as reported previously (Wang et al., 2016). Pregnancy was determined by observed estrous behaviors of surrogates at every ovulation cycle. After 150 days of pregnancy, newborn lambs were delivered and genotyped.

### Genotyping of Delivered Animals

Peripheral venous blood samples were collected from newborn lambs at day 15 after birth for genomic DNA extraction. Polymerase chain reaction (PCR) amplification-based Sanger sequencing was conducted using KOD-NEO-Plus enzyme (DR010A; TOYOBA, Osaka, Japan) and primers are listed in Supplementary Table S3.

### Prediction of Off-Target Sites

Potential off-target sites with up to three mismatches were predicted using the openly available tool SeqMap (Jiang and Wong, 2008). The process for searching for off-target sites was implemented as previously described (Wang et al., 2015; Niu et al., 2017). The primers used for amplifying off-target sites by captured deep sequencing are given in Supplementary Table S4.

### Captured Deep-Sequencing

On-target and potential off-target mutations were amplified using a KAPA HiFi HotStart PCR Kit (#KK2501; KAPA Biosystems, Wilmington, MA, United States) for deep sequencing library generation. Pooled PCR amplicons were sequenced using the MiSeq with TruSeq HT Dual Index system (Illumina, San Diego, CA, United States).

### Whole Genome Sequencing

Genomic DNA of nine animals from three edited families were used for WGS. Nine DNA libraries with insert sizes of approximately 300 bp were constructed following the manufacturer’s instructions, and 150-bp paired-end reads were generated using the Illumina HiSeq XTen PE150 platform. The qualified reads were mapped to the sheep reference genome (Jiang et al., 2014) using the BWA (v0.7.13) tool (Li and Durbin, 2009). Local realignment and base quality recalibration were assessed with the Genome Analysis Toolkit (GATK) (McKenna et al., 2010). Single-nucleotide polymorphisms (SNPs) and small indels (<50 bp) were called using GATK (McKenna et al., 2010) and SAMtools (Li et al., 2009).

### Identification of Off-Target Mutations

The called SNPs were filtered according to the following criteria: (1) SNPs that were identified by both GATK and SAMtools; (2) excluding SNPs that exist in NCBI sheep SNP database (>59 million SNPs); (3) excluding SNPs that exist in our sheep SNP database (*n* = 294, >79 million SNPs^1^); (4) within the remaining SNPs, SNPs with C and G converted to other base types were selected. The potential off-target sites were predicted using Cas-OFFinder (Bae et al., 2014) by consider allowing up to five mismatches. SNPs within the predicted off-target sites were identified as off-target mutations.

### Identification of *de novo* Mutations

Putative *de novo* SNPs and indels were identified according to our recent report (Wang et al., 2018). Briefly, the SNPs/indels were identified by both GATK and SAMtools, and SNPs/indels that were found in the NCBI and our own sheep SNP databases were removed. Next, the SNPs/indels inherited from parents were excluded. Additional SNPs/indels were filtered based on parameters including read depth and Phred-scaled likelihood (PL) scores (Wang et al., 2018). Finally, the mis-aligned or miscalled SNPs/indels were removed manually. Genome-wide structure variations (SVs) were called using BreakDancer (Chen et al., 2009), then the SVs specific in the edited animals were remained. To identify the *de novo* SVs, common SVs in every two founders, and the read depth <50%, as well as the scaffolds were removed.

## Results and Discussion

### Generation of Edited Animals

**FIGURE 1:**
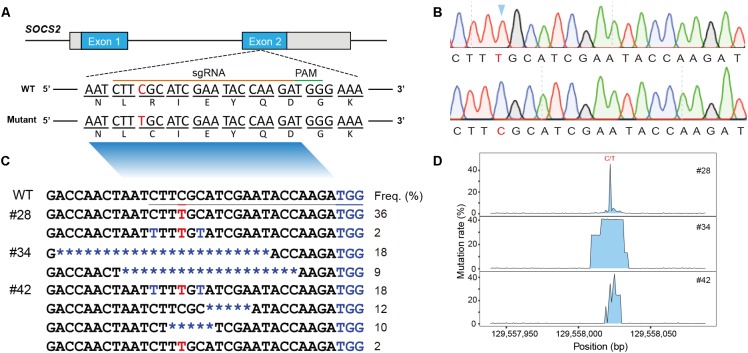
Animals and mutational spectra in the edited goat genome. **(A)** Scheme showing the target site in the sheep *SOCS2* gene. sgRNA sequences are presented in yellow. PAM sequences are highlighted in green. The BE3-mediated nucleotide substitutions (g.C1901T, p.R96C) are highlighted. **(B)** Sanger sequencing chromatogram of intended mutations directed by the BE3 system. **(C)** Genotypes of target sites derived from deep sequencing in the three founder animals. **(D)** Mutation rate at the targeted region.

To obtain lambs comprising the precise g.1901C > T mutation in *SOCS2*, we micro-injected the BE3 mRNA and sgRNA into the cytoplasm of 1-cell-stage embryos. The sgRNA was designed to encompass the target point mutation p.R96C in *SOCS2* (Figure 1A). Five mated Tan sheep that were treated for superovulation received 54 one-cell fertilized oocytes; after 53 embryos were subjected to micro-injection, 20 developing embryos were transplanted into eight recipients. Three recipient sheep were confirmed with pregnancy. After ∼150 days of gestation, four lambs (#28, #34, #41, and #42) were obtained (Table 1).

**Table 1:** Summary of the sheep generated with the defined point mutation via BE3.

Donor sheep	5
Collected embryos	54
BE3-sgRNA	
Injected embryos	53
Developing embryos	20
Recipient sheep	8
Gestation recipient	3
Newborns	4
Expected single base substitution	1
Indel	2
WT	1


Genomic DNA was isolated from the blood samples of the four lambs (#28, #34, #41, and #42) and the targeted region was evaluated by PCR-based Sanger sequencing; this analysis confirmed that three animals (#28, #34, and #42) were edited at the target site (Supplementary Figure S1A). We then used TA cloning and sequencing to further validate the genotypes of the three edited animals, and a nucleotide substitution at the p.R96C mutation site was found in #28 and #42 (Supplementary Figure S1B). TA-cloned sequencing further revealed short indels in the edited animals, for example, the founder animal #34 had 19- and 23-bp deletions and #42 was mosaic with the defined point mutation and a 5-bp deletion (Supplementary Figure S1B). To fully screen the genotypes in the edited animals, these three edited animals were subject to targeted deep sequencing, which confirmed the TA-sequencing results and identified additional low incidence of C-T genotypes within the editing window (Figure 1B,C; Gehrke et al., 2018). We demonstrated that BE3-medicated modification in sheep led to a gene knockout animal (#34), apparent mosaics, and a low incidence of short indels in edited animals (Figure 1D). To further investigate the mosaicism in the BE-edited animals, we sequenced the modified loci in additionally biopsied tissues (tail, muscle, and skin) of the three animals (#28, #34, and #42). We identified same heterozygous genotypes in these tissues as observed in whole blood in #28 and #42 (Supplementary Figure S1A), indicating the genetic modification occurred during early embryogenesis. The non-specificity of the programmable deaminase BE3 often results in short indels and mosaicism (Kim D. et al., 2017; Park et al., 2017; Sasaguri et al., 2018). Efforts have been made to optimize DNA specificity and minimize bystander effects of BEs (Rees et al., 2017; Gehrke et al., 2018) or to develop advanced cytidine and adenine BEs with high efficiency (e.g., BE4max, AncBE4max, and ABEmax; Koblan et al., 2018).

Although the editing efficiency in this study was as high as 75.0% (3/4), we only generated one animal with the precise point substitution (#28, 25%, 1/4) (Table 1). The efficiency of precise single-base substitution was equivalent to that observed in goats (24%) (Niu et al., 2018) and sheep (Zhou et al., 2018), and was significantly higher than that in zebrafish (4%) (Armstrong et al., 2016). We expect to use a variety of newly invented BEs to improve the DNA specificity and diminish bystander effects in the editing window.

### Phenotypes of Edited Animals

**FIGURE 2:**
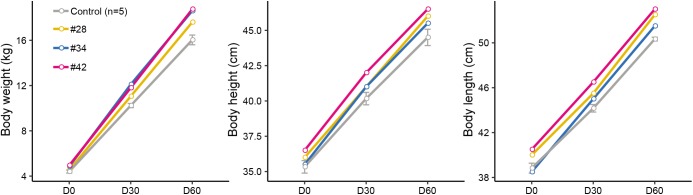
Observed morphological phenotypes in edited animals and controls. **(A)** Body weight. **(B)** Body height. **(C)** Body length.

Subsequently, we analyzed the growth curve and body size of mutant and control lambs to assess whether the p.R96C mutation impaired the function of the SOCS2 protein associated with morphology. The body weight of three edited sheep (#28, #34, and #42) was higher than that in the control group on D0, D30, and D60; body length and height in modified sheep were higher than those in the control group (Figure 2). We did not observe clear phenotypic differences in the three edited animals that were related to their genotypes (substitutions, deletions, or both), even the body parameters of #42 was higher than in the other two animals (Figure 2). Considering the mosaicism in the edited animals, more phenotypic data from a long period is needed to address the correlation of mutation types to phenotypes. Nevertheless, these results are consistent with a spontaneous mutation in *SOCS2* causing a 30–50% increase in the postnatal growth of mice (Horvat and Medrano, 2001), and the *SOCS2* p.R96C mutation in sheep led to an increase body weight, body size, and milk yield (Rupp et al., 2015). Moreover, *SOCS2* deletion protects bone heath in inflammatory bowel disease and causes a high-growth phenotype in mice (Horvat and Medrano, 2001; Dobie et al., 2018). We found that two animals (#34 and #42) with *SOCS2* indels were as healthy as normal sheep, and we did not observe any health issues.

### Off-Target Mutations in Edited Animals

**FIGURE 3:**
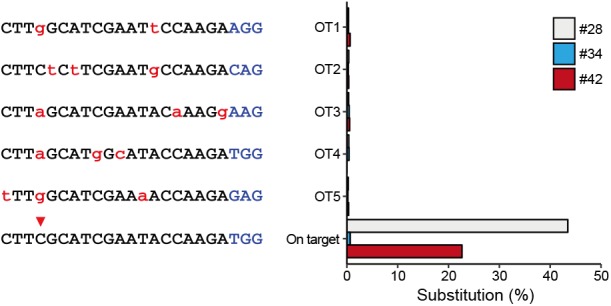
Detection of potential off-targeted sites by deep sequencing. Five potential off-targeted sites (OT1–OT5) were predicted by Cas-OFFinder. Deep sequencing was used to determine substitution frequencies at predicted target sites for the three founder animals. Mismatched nucleotide and PAM sequences are indicated in red and in blue, respectively.

To characterize off-target effects induced by the BE system, a deep sequencing assay was used to amplify predicted off-target sites in all the three edited animals. Five off-target sites (OT1–OT5) were predicted using the SeqMap tool (Jiang and Wong, 2008) (Supplementary Table S5). Targeted deep sequencing revealed that the frequency of BE3-induced point mutations is low at all predicted sites in all three founder animals (#28, #34, and #42) (Figure 3), indicating that the incidence of BE3-induced off-target mutations is rare.

**FIGURE 4:**
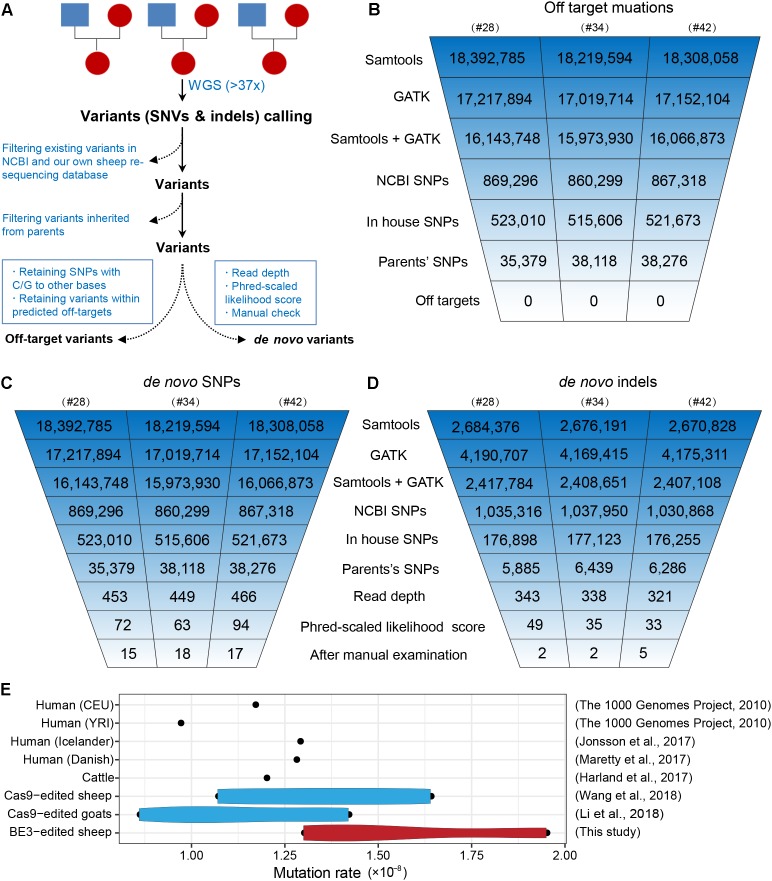
Identification of the genome-wide off-targets and *de novo* mutations by trio-based WGS. **(A)** Schematic representation of the study design for the identification of genome-wide off-target variants and *de novo* mutations. Summary of the filtering process of off-target mutations **(B)**, *de novo* SNPs **(C)**, and *de novo* indels **(D)**. **(E)** Mutation rates in BE3-edited sheep and other populations.

To further characterize off-target mutations at the whole-genome scale, we conducted family trio-based WGS to assess off-targets and *de novo* mutations in the three edited animals (#28, #34, and #42) (Figure 4A). We calculated the kinship coefficient for pair-wised animals to guarantee the pedigree information (Supplementary Table S6). The WGS yielded an average sequence coverage of 37.3× per individual, within a range of 34- to 41-fold, and generated 12–13 million SNPs for each animal (Supplementary Table S7). SNPs were first called by both GATK and SAMtools, and an average of 16 million SNPs were identified for each founder. Of the SNPs we were able to map in this study, we next removed naturally occurring variants in the NCBI sheep SNP database (>59 million SNPs) and in our own sheep SNP database (>79 million SNPs from 294 individuals) and filtered out SNPs that were inherited from parents, resulting in ∼37,000 remaining SNPs for each founder animal. We then excluded base substitutions including SNP types C to T/A/G and their antisense type G to A/T/C according to a recent study (Kim D. et al., 2017). Subsequently, we assessed the remaining SNPs that were within the predicted off-target sites (tolerant to five mismatches) (Supplementary Table S8) using Cas-OFFinder (Bae et al., 2014), and no single variants were identified (Figure 4B), indicating that no off-target mutations were induced by BE3 in the present study. The detailed filtering procedure is summarized in Supplementary Table S9.

To identify *de novo* mutations (SNPs and indels) in the edited animals, we used a stringent pipeline for variant filtering, as previously described (Li C. et al., 2018; Wang et al., 2018). Briefly, we selected SNPs that were identified by both GATK and SAMtools and removed existing SNPs in both the NCBI SNP database and our own sheep SNP database as well as the SNPs found in their parents. Next, we filtered out SNPs according to sequence read depth, PL scores, and manual examination of the FASTQ files (Wang et al., 2018). The remaining 15, 18, and 17 SNPs in individuals #28, #34, and #42 were identified as *de novo* SNPs for each progeny (Figure 4C). We next validated these *de novo* SNPs with Sanger sequencing. Of the 46 successfully amplified and sequenced SNPs, 44 of them were determined as true variants (Supplementary Figure S2 and Supplementary Table S10), indicating the pipeline for identification of *de novo* SNPs was robust. Similarly, we were able to identify 5, 5, and 10 *de novo* indels in individuals #28, #34, and #42, respectively (Figure 4D and Supplementary Table S11). To further characterize the large-scale genomic alterations induced by base editing, we called the SVs by BreakDancer (Chen et al., 2009), and identified a total of ten *de novo* SVs in the three BE-mediated animals (Supplementary Tables S12, S13), none of these variants were adjacent to the *SOSC2* site.

Additionally, we estimated the mutation rates per base pair per generation (Li C. et al., 2018), and found that no apparent differences between the BE3 and control animals in term of frequency of *de novo* SNPs in the present study and former studies (Figure 4E). Albeit only three trios were analyzed in this study, the mutation rate in base-edited sheep was equivalent to that in human populations (1000 Genomes Project Consortium et al., 2010; Maretty et al., 2017), cattle (Harland et al., 2017), as well as our previously generated CRISPR/Cas9-edited sheep and goat populations (Li C. et al., 2018; Wang et al., 2018). Along with our previous studies reporting the *de novo* mutations in edited animals and their offspring (Li C. et al., 2018; Li G. et al., 2018; Wang et al., 2018) and recently two trio-based studies in mice (Iyer et al., 2018; Willi et al., 2018), we demonstrate that the mutation frequency does not differ in Cas9-mediated or BE-mediated animals, thereby providing evidence to support the reliability of genome editing in large animals for biomedicine and agriculture.

## Conclusion

In summary, a single sheep carrying the *SOCS2* p.R96C mutation was successfully generated using programmable deaminases BE3. We confirmed that BE3 did not induce unintended off-target mutations at the genome-wide scale, and the mutation frequency in BE-mediated animals was equivalent to those in Cas9-edited animals and in natural populations. This study facilitates gene correction and genetic improvement of large animals caused by single base mutations.

## Data Availability

All relevant results are within the paper and its Supplementary Data Files. The raw WGS was available at NCBI SRA database under BioProject ID: PRJNA505205.

## Author Contributions

SZ, XZ, XW, BM, and YC conceived the study. BC, YW, QD, XZ, JL, YL, YD, GL, HY, and BM performed the experiments. CH, CL, and XW analyzed the dataset. QK and WN provided samples. XW, TS, and BP wrote the article.

## Conflict of Interest Statement

The authors declare that the research was conducted in the absence of any commercial or financial relationships that could be construed as a potential conflict of interest.

## Footnotes

1 http://animal.nwsuaf.edu.cn
