# Supplementary material for: Programmable Base Editing of the Sheep Genome Revealed No Genome-Wide Off-Target Mutations
Source: Front Genet. 2019 Mar 15;10:215. doi: 10.3389/fgene.2019.00215 (PMC6428697; doi:10.3389/fgene.2019.00215)
Supplement: Supplementary file 1 [file Data_Sheet_1.PDF]

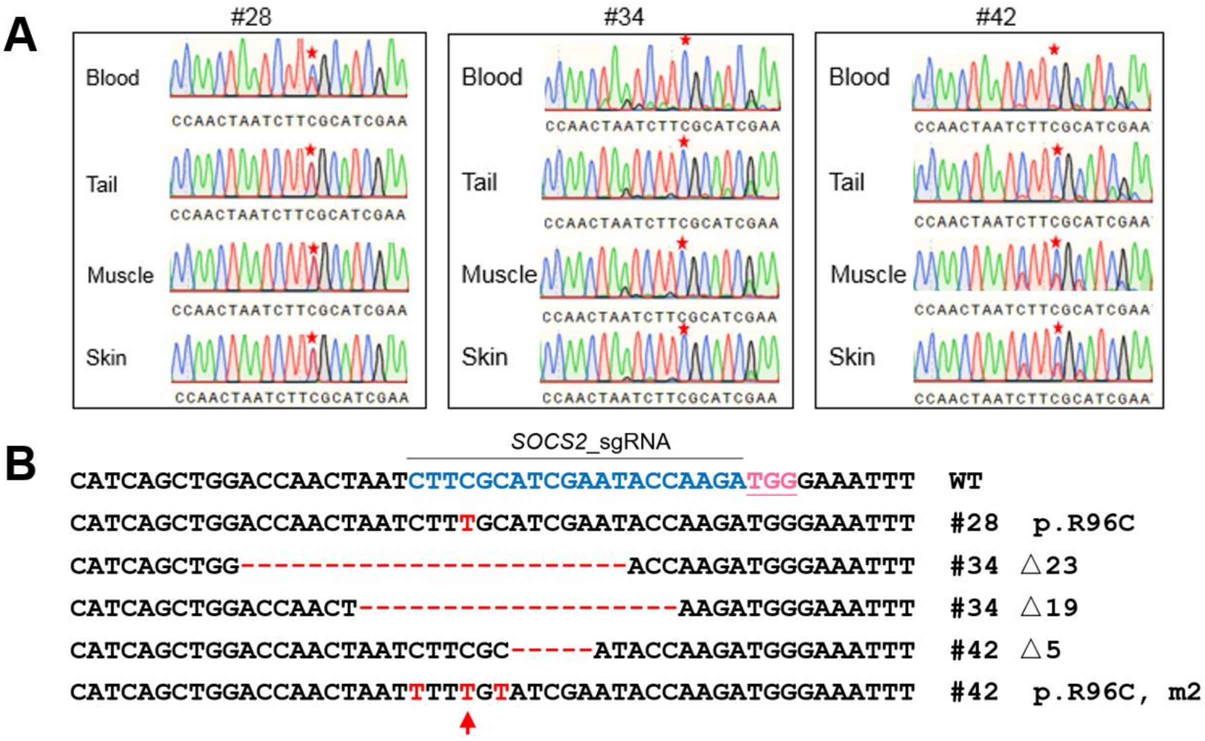

Supplementary Figure S1. (A) Genotypes of target sites in BE3-mediated animals in different tissues. **(B)** Sequencing alignment showing the genotypes of the mutated sites in three BE3-mediated animals. m2 indicates mutant at two additional sites.

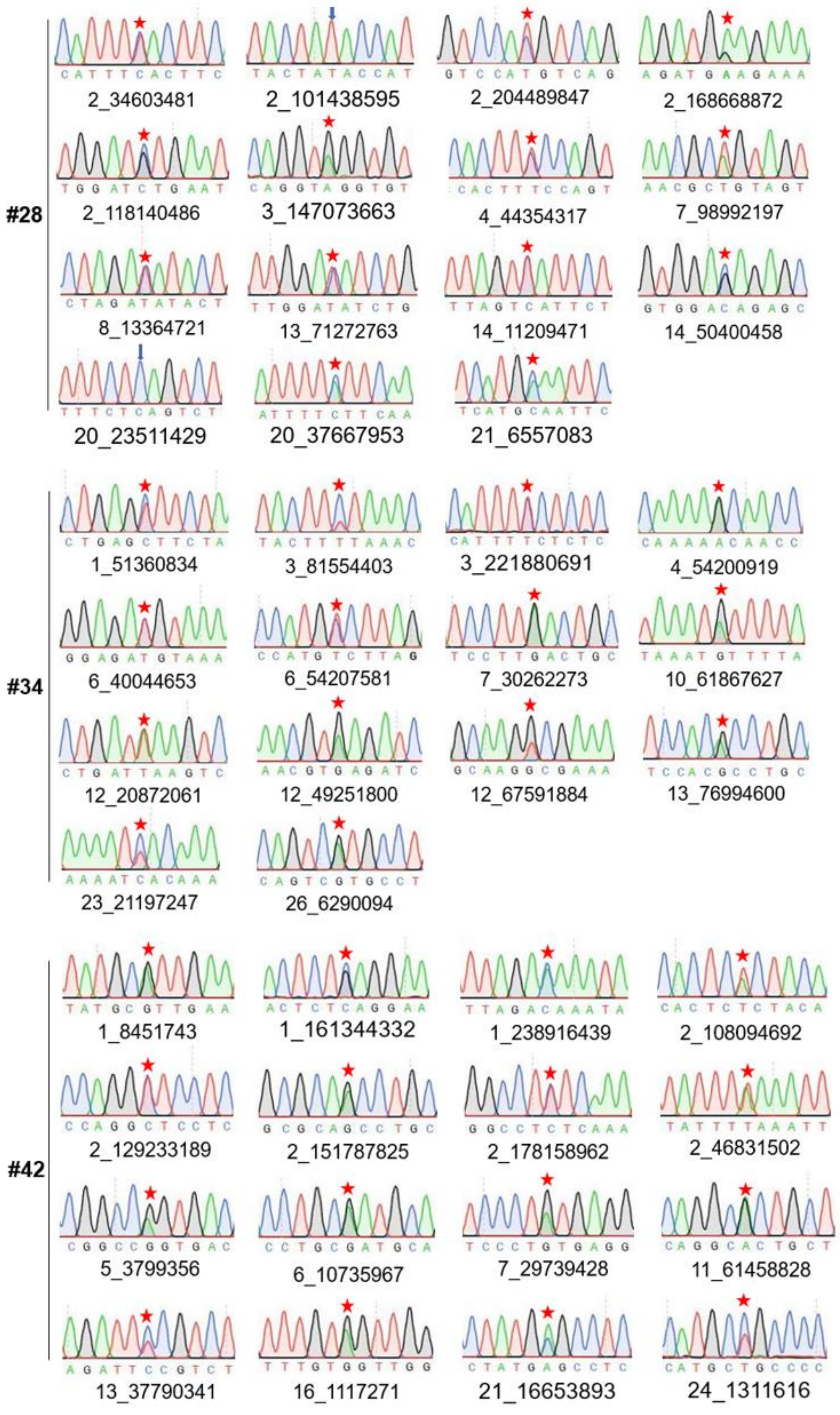

Supplementary Figure S2. Sanger sequencing validation of *de novo* SNPs in the BE-mediated animals. Asterisks represent true variants, while arrows indicate fake ones.
