# Supplementary material for: Programmable Base Editing of the Sheep Genome Revealed No Genome-Wide Off-Target Mutations
Source: Front Genet. 2019 Mar 15;10:215. doi: 10.3389/fgene.2019.00215 (PMC6428697; doi:10.3389/fgene.2019.00215)
Supplement: Supplementary file 2 [file Table_1.pdf]

Table S1 sgRNA of target sites.

**sgRNA**	**Targeting site**	**Location**	**Strand**
SOCS2 sgRNA	CTTCGCATCGAATACCAAGATGG	Chr3:129557939-129557961	+

Table S2 Oligonucleotides for generating sgRNA expression vectors.

**Name**	**Sequence**
SOCS2 sgRNA top strand	ACCGCTTCGCATCGAATACCAAGA
SOCS2 sgRNA bottom strand	AAACTCTTGGTATTCGATGCGAAG

Table S3 Primers for genotyping and amplifying BE3/sgRNA targeted region.

**Name**	**Sequence**	**Amplicon (bp)**
SOCS2_F	TTCAGAAAATTAAACGGACCC	437
SOCS2_R	TAGTAGTCGATCAGATGAACC	

Table S4 Primers for genotyping and amplifying predicted off-target site fragments.

**Off-target site**	**Sequence**	**Amplicon (bp)**
SOCS2 -OT1F	AATATTTTTGCGTGTGGACAG	242 bp
SOCS2 -OT1R	CAATTCCCTCACACTACAGTT	
SOCS2 -OT2F	GATTTAGCTTGTGTTTCTTCC	289 bp
SOCS2 -OT2R	GAGAATGTCAAAGCCCTTAAC	
SOCS2 -OT3F	GTTTTCTTTGGTTAGGTCTGC	372 bp
SOCS2 -OT3R	CTGATTGGTTAGTTCCTGTGC	
SOCS2 -OT4F	CCCTACTGTGACTGCTTTCCA	347 bp
SOCS2 -OT4R	ATGTTCCTAGCCACTACTGAT	
SOCS2 -OT5F	TCACACTGATGAGGAAAGGTC	280 bp
SOCS2 -OT5R	ACCTTTGCAACTTTCATCACA	

Table S5 List of predicted off-target sites.

Position	20	19	18	17	16	15	14	13	12	11	10	9	8	7	6	5	4	3	2	1	N	G	G	Location			
sgRNA	C	T	T	C	G	C	A	T	C	G	A	A	T	A	C	C	A	A	G	A	T	G	G	Chr.	Start	End	Strand
OT1	C	T	T	G	G	C	A	T	C	G	A	A	T	T	C	C	A	A	G	A	A	G	G	17	18,979,452	18,979,474	–
OT2	C	T	T	C	T	C	T	T	C	G	A	A	T	G	C	C	A	A	G	A	C	A	G	11	47,007,787	47,007,809	+
OT3	C	T	T	A	G	C	A	T	C	G	A	A	T	A	C	A	A	A	G	G	A	A	G	15	78,397,001	78,397,023	+
OT4	C	T	T	A	G	C	A	T	G	G	C	A	T	A	C	C	A	A	G	A	T	G	G	5	79,633,962	79,633,984	–
OT5	T	T	T	G	G	C	A	T	C	G	A	A	A	A	C	C	A	A	G	A	G	A	G	6	22,710,538	22,710,560	–
